# Extended depth of focus IOL in eyes with different axial myopia and targeted refraction

**DOI:** 10.1186/s12886-024-03442-5

**Published:** 2024-04-22

**Authors:** Xiaohui Wang, Sinan Liu, Yinqi Chen, Jinping Gong, Nuozhou Wu, Yihua Yao

**Affiliations:** 1 Department of Ophthalmology, The First Affiliated Hospital of Fujian Medical University, 20 Cha Zhong Road, 350005 Fujian, Fujian China; 2 Department of Ophthalmology, Binhai campus of the First Affiliated Hospital, National Regional Medical Center, Fujian Medical University, Fujian, China; 3 Fujian Institute of Ophthalmology, The First Affiliated Hospital of Fujian Medical University, Fuzhou, Fujian China; 4 Fujian Provincial Clinical Medical Research Center of Eye Diseases and Optometry, The First Affiliated Hospital of Fujian Medical University, Fuzhou, Fujian China

**Keywords:** Extended depth of focus, Intraocular lens, High myopia, Axial length, Myopic target, Visual results, Cataract surgery

## Abstract

**Aim:**

To evaluate the objective visual outcomes following implantation of extended depth of focus intraocular lens (EDOF IOL) in individuals with varying axial lengths (AL) and targeted refraction.

**Methods:**

This retrospective study comprised age-matched eyes that underwent implantation of the EDOF IOL. Eyes were categorized based on AL into groups: control group with AL < 26 mm; high myopia group with AL ≥ 26 mm. Each group was then subdivided based on postoperative spherical equivalent (SE). Follow-up at three months included assessment of uncorrected visual acuity at different distances, contrast sensitivity (CS), refractive outcomes, and spectacle independence.

**Results:**

Overall, this study included 100 eyes from 100 patients, comprising 50 males (50.00%) and 50 females (50.00%), with 20 eyes in each group. In the control group, the uncorrected distance visual acuity (UDVA) at 5 and 3 m (m) in the − 1.50 to -0.75 group was inferior to that of the − 0.75 to 0.00 group (*P* = 0.004). Conversely, the uncorrected near visual acuity (UNVA) at 33 cm in the − 1.50 to -0.75 group was superior to that of the − 0.75 to 0.00 group (*P* = 0.005). Within the high myopia group, the UDVA at 5 and 3 m in the − 2.25 to -1.50 group was worse than in the − 0.75 to 0.00 group (*P* = 0.009 and 0.008, respectively). However, the UNVA at 33 cm in the − 2.25 to -1.50 group was better than in the − 0.75 to 0.00 group (*P* = 0.020). No significant differences were observed among the groups for corrected distance visual acuity (CDVA) (*P* > 0.05). Additionally, in the high myopia group, the CS of the − 2.25 to -1.50 group was lower compared to that of the − 0.75 to 0.00 group (*P* = 0.017). Among high myopia patients, 90.00% with refraction ranging from − 1.50 to -0.75 reported achieving overall spectacle independence.

**Conclusions:**

Implantation of extended depth of focus intraocular lenses (IOLs) yields satisfactory visual and refractive outcomes in eyes with axial myopia. Among high myopia patients, a refraction ranging from − 1.50 to -0.75 diopters achieves superior visual quality compared to other postoperative myopic diopters.

## Introduction

With the advancements in refractive cataract surgery and the widespread adoption of presbyopia-correcting IOLs [1], patients with cataracts now have a broader range of options available. These options promise to provide spectacle independence across various distances, including far, intermediate, and near vision [2, 3]. Various presbyopia-correcting IOLs, such as multifocal IOLs and extended depth of focus intraocular lenses(EDOF IOLs), are available to fulfill visual needs at different distances [4, 5]. Multifocal intraocular lenses (MIOLs), incorporating diffractive optics, provide patients with enhanced vision for both near and far distances when compared to monofocal IOLs [6]. However, postoperative visual optical disturbances associated with multifocal IOLs make it difficult for patients. Moreover, given the elevated expense associated with trifocal IOLs, EDOF IOLs are occasionally favored by both doctors and patients. EDOF IOLs employ diffractive echelette technology to extend a single constant focal point into a continuous transitional focus line [7–9]. This design allows for minimal impact on retinal image quality loss, and comprising the tolerance of slight refractive errors. Additionally, EDOF IOLs effectively mitigate the halo effect commonly associated with multifocal IOLs, thereby enhancing perceived retinal image quality.

High myopia combined with cataracts (HMC) significantly impacts the quality of life for affected individuals, who often rely on thick-framed glasses for daily activities and work over the long term. Therefore, attaining spectacle independence or even needing only low-powered corrective glasses post-surgery would markedly enhance the postoperative satisfaction of this population, liberating them from the burden of cumbersome eyewear. Implantation of multifocal IOLs in HMCs has demonstrated favorable clinical outcomes [10–13]. The study [14] involving the implantation of trifocal IOLs in highly myopic patients categorized by AL demonstrated that trifocal IOLs implanted in HMC provided similar visual outcomes to those of non-myopic cataract patients. However, there is limited research available regarding postoperative visual quality in individuals with high myopia combined with cataracts (HMC) who undergo implantation of EDOF IOLs. Therefore, this study conducted a retrospective, non-randomized clinical investigation to evaluate the visual quality of HMC patients who underwent EDOF IOL implantation, with a follow-up period of at least three months. The study reports on postoperative uncorrected and corrected visual acuity, CS, refractive outcomes and overall spectacle independence.

## Methods

### Patients and intraocular lenses

This retrospective investigation was established to assess the surgical outcomes associated with age-matched cataract patients who underwent implantation of EDOF IOLs (The TECNIS Symfony IOL, Johnson & Johnson Vision) across various postoperative refraction profiles. In agreement with earlier studies [15], the eyes were separated and built on their ALs. The first contained AL < 26 mm (titled control group), and the second included AL ≥ 26 mm (titled high myopia group). Additionally, the control group was subdivided into the − 0.75 to 0.00 and − 1.50 to -0.75 groups, while the high myopia group was further divided into the − 0.75 to 0.00, -1.50 to -0.75, and − 2.25 to -1.50 groups. The research was conducted in accordance with the ethical standards outlined by the Ethics Committee of the First Affiliated Hospital of Fujian Medical University and adhered to the principles of the 1964 Helsinki Declaration and its subsequent amendments.

Patients included in the study were between 18 and 80 years old, with corneal astigmatism of less than 1.50 diopters (D), and a Kappa angle no greater than 0.5 mm. Eyes with complications that could affect visual performance, such as macular pathology, uncontrolled glaucoma, or zonular or capsular frailties, were excluded from the study. Additionally, patients who did not attend postoperative visits or who had undergone other ocular surgeries, including laser therapies, were also excluded. Finally, the 100 eyes included in the study were divided into five groups of 20 (20.00%) each based on their AL and postoperative SE.

### Preoperative examinations

The changes in the fundus were captured using ultra-wide field retinal imaging or scanning laser ophthalmoscopy (SLO, Optos Inc) and optical coherence tomography (OCT, Carl Zeiss Meditec). The Kappa angle was measured by corneal topography, Pentacam (Oculus Optikgeräte GmbH). Moreover, optical biometry, such as the steep and flap radius curvature of the cornea, corneal astigmatism, anterior chamber depth (ACD), and AL, were acquired by Lenstar 900 (Haag-Streit AG).

### IOL power calculation and refractive target strategy

The Barrett Universal II formula was utilized to calculate the intraocular lens (IOL) power using the data obtained from the Lenstar 900. The residual nearsighted power was typically targeted at -0.75 to 0.00, -1.50 to -0.75, and − 2.25 to -1.50 to mitigate the onset of presbyopia-induced near-focus distance drift [16]. The surgeon also predicated on the earlier surgical experience, the patient’s preoperative expectancy, and postoperative all-range visual outcomes to select preferred IOL degrees. Due to the increased cost associated with toric extended depth of focus (EDOF) intraocular lenses (IOLs), we opted for bilateral symmetrically placed limbal relaxing incisions positioned in the steep meridian of corneal astigmatism, as calculated by the Barrett Toric calculator, to address any residual astigmatism. Paired opposite transparent corneal incisions were created in patients with > 0.75 D preoperative cornea astigmatism (*n* = 40, 44.00%). Using preoperative corneal curvature measurements obtained from Pentacam and Lenstar 900, the Barrett Toric Calculator, linked with residual corneal astigmatism, was employed to determine the locations for symmetrical limbal incisions.

### Surgical technique

Generally, the standard refractive cataract surgery procedure lasted for 15 minutes. Corneal tunnel incisions of 2.4 mm were symmetrically created based on the preoperatively calculated axial position of astigmatism. Continuous curvilinear capsulorhexis (CCC) was performed at 5.5 mm diameter, centered in the middle of the anterior capsule. Followed by the operation of phacoemulsification (Infiniti System, Alcon) and embedded with a Symfony IOL. Before irrigation and aspiration, the surgeon meticulously polished the cortex on the posterior capsular bag to mitigate the development of posterior capsular opacification (PCO) post-surgery.

### Postoperative examinations

At three months or more post-surgery, uncorrected and distance-corrected visual acuities at various distances including 5 m, 3 m, 1 m, 60 cm, 40 cm, and 33 cm (UDVA, CDVA at 5 m, UDVA at 3 m, UIVA at 1 m, UIVA at 60 cm, UNVA at 40 cm, and UNVA at 33 cm) were assessed using the Binoptometer 4P (Oculus Optikgerate GmbH) and recorded in logarithms of the minimal angle of resolution (logMAR). Furthermore, contrast sensitivity (CS) tests were conducted using the Binoptometer 4P under photopic conditions (85 cd/m2) at a distance of 3 m. Postoperative spherical equivalent (SE), spherical, and cylinder magnitudes were evaluated using the Digital Phoropter (Huvitz) to obtain precise measurements of the residual nearsighted power. Information regarding overall spectacle independence was gathered by asking patients whether they required spectacles for far, intermediate, or near vision.

### Statistical analysis

All clinical parameters were analyzed using SPSS 25.0 (version 25, IBM Corp.). One-way analysis of variance (ANOVA) with Bonferroni’s adjustment was utilized to compare parametric variables among groups. For non-parametric variables, the Kruskal-Wallis test was employed. And categorical variables were compared using the χ^2^ test. Graphs depicting the research trends were generated using GraphPad Prism 9. Statistical significance was determined when the *P*-value was less than 0.05 (*P* < 0.05).

## Results

The current study included 100 eyes from 100 patients, with an equal distribution of 50 males (50.00%) and 50 females (50.00%). The AL ranged from 23 to 31 mm, and data collection spanned from 2020 to 2023. Postoperative follow-up assessments were conducted at three months after surgery. Based on different axial lengths (ALs), two age-matched groups were created: the control group (AL < 26 mm) and the high myopia group (AL ≥ 26 mm). The mean age of the study population was 56.09 ± 12.39 years. Table 1 described the control group’s preoperative demographic features and optical biometry. There were no differences from basis data between postoperative SE groups (entirely *P* > 0.05), apart from IOL power and target SE (*P* = 0.014 and 0.001). As depicted in Table 2, there were no significant differences in preoperative baseline data among the postoperative spherical equivalent (SE) groups (*P* > 0.05 for all comparisons), except for target SE (*P* = 0.001).

**Table 1:** Preoperative demographic features with the control group eyes

Parameter	[-0.75, 0.00)	[-1.50, -0.75)	P
Age (years)	58.33 ± 15.21	48.42 ± 16.38	0.089
AL (mm)	24.51 ± 0.88	24.98 ± 0.84	0.140
Corneal astigmatism (D)	1.05 ± 1.64	1.38 ± 0.97	0.560
ACD (mm)	3.40 ± 0.43	3.38 ± 0.40	0.910
R_1_ (mm)	7.79 ± 0.23	7.67 ± 0.20	0.193
R_2_ (mm)	7.31 ± 1.66	7.44 ± 0.24	0.814
Kappa angle (mm)	0.19 ± 0.09	0.21 ± 0.18	0.643
Uncorrected visual acuity (logMAR)	0.64 ± 0.33	0.64 ± 0.38	0.960
IOL power (D)	18.83 ± 2.79	15.79 ± 3.88	0.014^*^
Target SE (D)	-0.56 ± 0.25	-0.82 ± 0.13	0.001^*^

*: Significant difference between groups.Student’s t tests are indicated as mean ± SD.

AL = axial length, SD = standard deviation, D = diopter, ACD = anterior chamber depth, R_1_ = flat axial corneal curvature, R_2_ = steep axial of corneal curvature, logMAR = logarithm of the minimum angle of resolution, IOL = intraocular lens, SE = spherical equivalent.

**Table 2:** Preoperative demographic features with the high myopia group eyes

Parameter	[-0.75, 0.00)	[-1.50, -0.75)	[-2.25, -1.50)	P
Age (years)	58.65 ± 9.45	53.95 ± 9.43	53.71 ± 8.60	0.268
AL (mm)	27.83 ± 1.36	27.91 ± 1.47	28.67 ± 1.80	0.433
Corneal astigmatism (D)	0.90 ± 0.61	1.02 ± 0.73	1.24 ± 0.66	0.533
ACD (mm)	3.41 ± 0.31	3.40 ± 0.40	3.50 ± 0.32	0.788
R_1_ (mm)	7.77 ± 0.22	7.74 ± 0.20	7.65 ± 0.20	0.422
R_2_ (mm)	7.62 ± 0.24	7.57 ± 0.26	7.52 ± 0.22	0.683
Kappa angle (mm)	0.26 ± 0.31	0.24 ± 0.13	0.23 ± 0.13	0.938
Uncorrected visual acuity (logMAR)	0.78 ± 0.41	0.75 ± 0.36	0.83 ± 0.42	0.879
IOL power (D)	10.68 ± 3.89	10.08 ± 3.23	8.36 ± 4.50	0.385
Target SE (D)	-0.97 ± 0.89	-1.37 ± 0.86	-2.28 ± 1.24	0.015^*^

^*^: Significant difference among three groups.

One-way ANOVA tests or Kruskal-Wallis are indicated as mean ± SD.

AL = axial length, SD = standard deviation, D = diopter, ACD = anterior chamber depth, R_1_ = flat axial corneal curvature, R_2_ = steep axial of corneal curvature, logMAR = logarithm of the minimum angle of resolution, IOL = intraocular lens, SE = spherical equivalent.

**Fig. 1:**
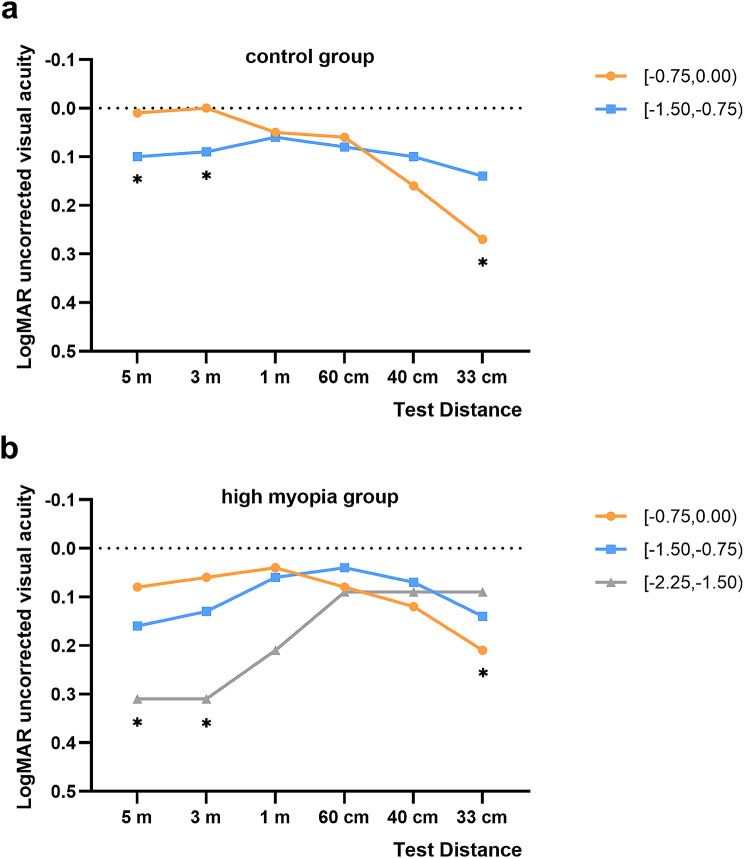
**a**: Mean uncorrected visual acuities at 5 m, 3 m, 1 m, 60 cm, 40 cm, and 33 cm among groups with the control group eyes. **b**: Mean uncorrected visual acuities at 5 m, 3 m, 1 m, 60 cm, 40 cm, and 33 cm among groups with the high myopia group eyes. *: *P* < 0.05. logMAR = logarithm of the minimum angle of resolution

Figure 1a illustrated the uncorrected visual acuity after implantation of EDOF IOL at the three-month follow-up. In the control group, the uncorrected distance visual acuities at 5 m and 3 m for the − 1.50 to -0.75 group were significantly inferior compared to the − 0.75 to 0.00 group (refer to Table 3, *P* = 0.004 and 0.004, respectively). In contrast, the uncorrected 33 cm near visual acuity of the − 1.50 to -0.75 group was significantly better than the − 0.75 to 0.00 group (Table 3, *P* = 0.005). No differences were found in 1 m, 60 cm, and 40 cm UDVA and CDVA between the − 1.50 to -0.75 group and the − 0.75 to 0.00 group (Table 3, all *P* > 0.05).

**Table 3:** Postoperative different test distance visual acuities (logMAR) with the control group eyes

Parameter	[-0.75, 0.00)	[-1.50, -0.75)	P
UDVA (5 m)	0.01 ± 0.07	0.10 ± 0.08	0.004^*^
UDVA (3 m)	0.00 ± 0.07	0.09 ± 0.09	0.004^*^
UIVA (l m)	0.05 ± 0.07	0.06 ± 0.10	0.627
UIVA (60 cm)	0.06 ± 0.08	0.08 ± 0.10	0.585
UNVA (40 cm)	0.16 ± 0.11	0.10 ± 0.12	0.117
UNVA(33 cm)	0.27 ± 0.12	0.14 ± 0.11	0.005^*^
CDVA (5 m)	-0.03 ± 0.08	-0.04 ± 0.07	0.778
SE (D)	-0.28 ± 0.34	-1.21 ± 0.21	<0.001^*^
PE (D)	0.38 ± 0.30	0.38 ± 0.29	0.983

^*^: Significant difference between groups.

*P* values less than 0.05 are considered statistically significant.

Student’s t tests are indicated as mean ± SD.

logMAR = logarithm of the minimum angle of resolution, UDVA = uncorrected distance visual acuity, UIVA = uncorrected intermediate visual acuity, UNVA = uncorrected near visual acuity, logMAR = logarithm of the minimum angle of resolution, CDVA = corrected distance visual acuity, SE = spherical equivalent, PE = prediction error, D = diopter.

Figure 1b compared the visual acuities among the three postoperative SE groups in the high myopia group. The uncorrected distance visual acuities at 5 m and 3 m for the − 2.25 to -1.50 group were significantly poorer compared to the − 0.75 to 0.00 group (refer to Table 4, *P* = 0.009 and 0.008, respectively). Conversely, the uncorrected near visual acuity at 33 cm for the − 2.25 to -1.50 group was significantly better than the − 0.75 to 0.00 group (refer to Table 3, *P* = 0.020). There were no significant differences in uncorrected visual acuities at other distances and CDVA among the three different postoperative SE groups in the high myopia group (Table 4, all *P* > 0.05).

**Table 4:** Postoperative different test distance visual acuities (logMAR) with the high myopia group eyes

Parameter	[-0.75, 0.00)	[-1.50, -0.75)	[-2.25, -1.50)	P
UDVA (5 m)	0.08 ± 0.08	0.16 ± 0.12	0.31 ± 0.24	0.009 ^a^
UDVA (3 m)	0.06 ± 0.08	0.13 ± 0.10	0.31 ± 0.25	0.008 ^a^
UIVA (l m)	0.04 ± 0.08	0.06 ± 0.07	0.21 ± 0.20	0.082
UIVA (60 cm)	0.08 ± 0.10	0.04 ± 0.06	0.09 ± 0.10	0.315
UNVA (40 cm)	0.12 ± 0.11	0.07 ± 0.09	0.09 ± 0.10	0.288
UNVA(33 cm)	0.21 ± 0.11	0.14 ± 0.10	0.09 ± 0.04	0.020 ^a^
CDVA (5 m)	-0.01 ± 0.09	-0.03 ± 0.05	-0.04 ± 0.05	0.552
SE (D)	-0.57 ± 0.29	-1.29 ± 0.20	-2.21 ± 0.53	<0.001^*^
PE (D)	0.55 ± 0.77	0.38 ± 0.83	0.57 ± 0.42	0.754

^*^: Significant difference among three groups.

^a^: Significant difference between [-0.75,0.00) group and [-2.25, -1.50) group.

*P* values less than 0.05 are considered statistically significant.

One-way ANOVA tests are indicated as mean ± SD.

logMAR = logarithm of the minimum angle of resolution, UDVA = uncorrected distance visual acuity, UIVA = uncorrected intermediate visual acuity, UNVA = uncorrected near visual acuity, logMAR = logarithm of the minimum angle of resolution, CDVA = corrected distance visual acuity, SE = spherical equivalent, PE = prediction error, D = diopter.

The control group showed that the 3-month postoperative SE distribution differs significantly (Table 3, -0.28 ± 0.34 D versus − 1.21 ± 0.21 D; *P* < 0.001). Similarly, in the high myopia group, postoperative SE showed statistically significant differences for all comparisons (refer to Table 4, -0.57 ± 0.29 D versus − 1.29 ± 0.20 D versus − 2.21 ± 0.53 D, *P* < 0.001). This indicates that the amount of actual reserved nearsighted power of the EDOF IOL differed among the distinct postoperative SE groups. The variance between postoperative and preoperative reserved target myopic diopters corresponds to the prediction error (PE). No statistically significant among-group differences emerged in the PE (Tables 3 and 4, *P* = 0.983 and 0.754).

**Fig. 2:**
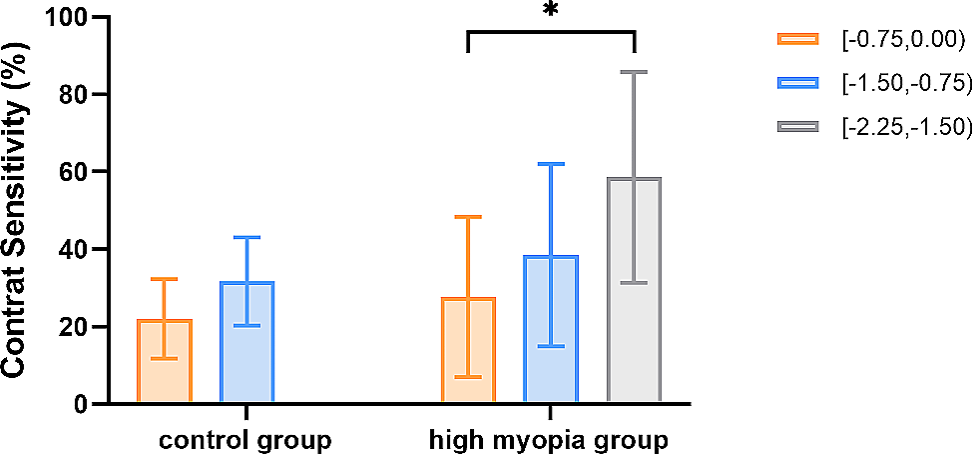
Contrast sensitivity of five postoperative SE groups. *: *P* < 0.05

Figure 2 illustrated the differences in uncorrected visual acuity CS under photopic conditions among the groups. Patients achieved better photopic CS when they were able to perceive more characters in backgrounds with reduced contrast. The CS detected under uncorrected visual acuity of the − 2.25 to -1.50 group was significantly worse than that of the − 0.75 to 0.00 group in the high myopia group (58.57 ± 27.19 D versus 27.65 ± 20.70 D; *P* = 0.017). Furthermore, no significant difference in CS was found between the two postoperative SE groups in the control group (*P* = 0.061).

**Fig. 3:**
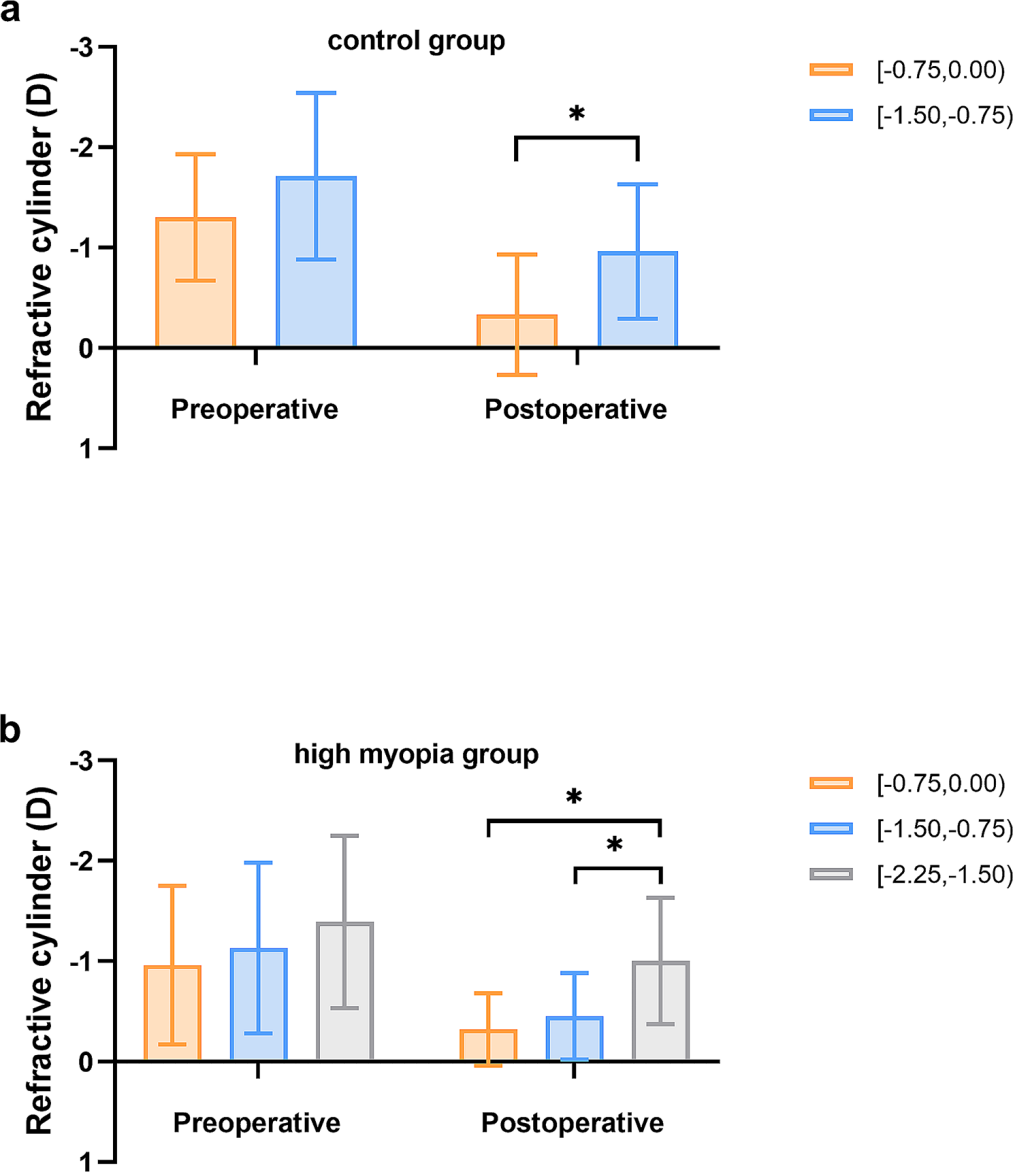
**a**: Refractive cylinder of preoperative and postoperative outcomes among groups with the control group eyes. **b**: Refractive cylinder of preoperative and postoperative outcomes among groups with the high myopia group eyes. *: *P* < 0.05. D = Diopter

Figure 3 indicated that the distribution of preoperative refractive cylinder among postoperative SE groups in the two ALs groups did not differ significantly (Tables 1 and 2, *P* = 0.560 and 0.533). However, the 3-month postoperative refractive cylinder was significantly better in the − 0.75 to 0.00 group compared to the − 1.50 to -0.75 group in the control group (-0.33 ± 0.60 versus − 0.96 ± 0.67; *P* = 0.010). In the high myopia group, the − 0.75 to 0.00, -1.50 to -0.75, and − 2.25 to -1.50 groups of refractive cylinder varied from 0.90 ± 0.61, 1.02 ± 0.73, and 1.24 ± 0.66 preoperatively to -0.32 ± 0.36, -0.45 ± 0.43 and − 1.00 ± 0.63 obtained at three-month follow-up, respectively. Furthermore, the 3-month postoperative refractive cylinder was significantly worse in the − 2.25 to -1.50 group compared to the − 0.75 to 0.00 and − 1.50 to -0.75 groups among the high myopia (*P* = 0.011).

After surgery, 65.00% (13 of 20 patients) in the − 0.75 to 0.00 refractive control group and 80.00% (16 of 20 patients) in the − 1.50 to -0.75 refractive control group achieved overall spectacle independence (χ^2^ test, *P* = 0.087). In the high myopia group, with a target of -0.75 to 0.00, 11 of 20 patients 55.00%, with a target of -1.50 to -0.75, 18 of 20 patients 90.00% and with a target of -2.25 to -1.50, 10 of 20 patients 50.00% obtained overall spectacle independence. The rates of overall spectacle independence in the high myopia group with a target of -1.50 to -0.75 showed significantly better results than that of other refractive groups (χ^2^ test, *P* = 0.047).

## Discussion

We assessed the visual performance of cataract patients implanted with EDOF IOLs at a 3-month follow-up, considering different ALs and distinct postoperative SE values. The study concluded that the ALs did not impact the visual quality within the same postoperative SE group. In contrast, the postoperative SE did influence the visual outcomes within the same AL group. Additionally, we observed that the eyes in both the control and high myopia groups demonstrated favorable visual and refractive outcomes. In the control group, the distance visual results with a target of -1.50 to -0.75 were not as good as that of -0.75 to 0.00 diopters. Additionally, the group’s results with a target of -1.50 to -0.75 exhibited better visual quality than other SE groups in the high myopia eyes.

According to recent research, our current study represents the first clinical design involving two distinct ALs and three separate refractive diopters for implantation with EDOF IOLs, predominantly focusing on patients with axial myopia eyes. Previous studies mainly consisted of clinical observational cases in such patients. Additionally, we employed an individual matching method based on patient age and other similar features, thereby reducing statistical bias in the demographics among groups.

The current study found good visual acuities at different distances after implantation of EDOF IOL in the high myopia eyes; the uncorrected distance visual acuities measured at 5 m and 3 m are statistically favorable in the − 0.75 to 0.00 group than those in the − 1.50 to -0.75 or -2.25 to -1.50 groups. The statistically superior uncorrected near visual acuities at 33 cm observed in the − 2.25 to -1.50 or -1.50 to -0.75 groups compared to the − 0.75 to 0.00 group could be attributed to the higher targeted refraction in the − 2.25 to -1.50 myopic reserved group preoperatively. Meng et al. stratified their patients based on AL into three groups: below 26.0 mm, 26.0 to 28.0 mm, and above 28.0 mm. They implanted a trifocal IOL and observed similar visual outcomes in eyes within the − 0.75 to 0.00 group (both high myopic and non-myopic eyes). Bai et al. [17] reported that the uncorrected visual acuity in patients with an axial length of ≥ 24.5 mm was 0.09 ± 0.08 at distance and 0.14 ± 0.08 at near vision. Sandoval et al. [18] revealed that the nondominant eye was targeted slightly myopic in the mini-monovision group, which provided improved near visual acuity. These outcomes closely align with those observed in the high myopia group of our study, which could be attributed to the diverse levels of near vision preserved preoperatively. Besides, we found that reflected from the similar CDVA among groups, EDOF IOLs offered reliable efficacy for the eye with various postoperative residual myopic refraction, even for the eyes targeted between − 2.25 to -1.50. Sun [19] and Steinwender [14] emphasized the superior corrected distance visual acuity achieved with the diffractive trifocal IOL. Sun et al. [20] reported that trifocal IOL offered stable and satisfactory visual outcomes for eyes with long AL. Thus, our research confirmed that the macular function of patients appeared not to be defective.

In this study, the between-group contrast sensitivities of the control group were similar. However, the CS of the − 2.25 to -1.50 group was significantly worse than that of the − 0.75 to 0.00 group in the high myopia group, indicating that the improvement in CS in the high myopia group is supported by the distance vision recorded at 3 m, consistent with reports by Schallhorn et al. and Chen et al. [21, 22]. Several previous reports addressed that the macular function was unimpaired and longer AL eyes still can provide good visual quality [10, 11, 23]. The level of CS is primarily influenced by the postoperative SE and also depends on the potential uncorrected visual acuity. Therefore, the observed poorer CS in the − 2.25 to -1.50 group might be attributed to a design with more near attached powers related to the preoperative target SE. However, high myopia patients targeted from − 1.50 to 0.00 still achieve good CS after surgery.

We found that there were no statistically significant differences among groups in terms of PE, suggesting that measurement errors caused by the optical evaluation devices could be considered negligible. Another reason for the accuracy of this measurement data is the absence of posterior staphyloma or myopic maculopathy in elongated eyeballs [24]. The optimized “A” constant and the Barrett Universal II formula were used to calculate the magnitude of the intended implantation IOLs [25]. Cheng et al. [26] addressed Holladay 1 with Wang-Koch adjustment had better accuracy than Barrett for ALs between 25.0 mm and 27.0 mm, but this study had not been published when our trial started. The previous studies [27–30] were comparable to the above-reported analyses.

In the present study, the mean reduction in corneal astigmatism magnitude ranged from 0.50 D to 2.25 D, achieved through the use of paired 2.4 mm corneal tunnel incisions. This approach significantly improved preexisting corneal astigmatism, with none of the eyes exhibiting residual astigmatism exceeding 2.0 D after surgery. These findings are consistent with recently published statistics [31–33]. Additionally, our results indicate that corneal astigmatism exhibited similar outcomes between the − 1.50 to -0.75 and − 0.75 to 0.00 refractive diopter groups following surgery. Mendicute et al. [34] addressed that paired opposite clear corneal incisions (OCCIs) and toric IOL implantation were reliable and effective alternatives for treating preexisting astigmatism. Ren et al. [35] reported a mean symmetric reduction in astigmatism of 0.61 D with the use of the 3.0 mm OCCIs method, without causing additional corneal aberrations. In summary, for eyes with preexisting corneal astigmatism greater than 0.75 D and when toric EDOF IOLs were not available, adopting opposite clear tunnel corneal incisions is a prudent option.

Here, we achieved satisfactory spectacle independence for all ranges of vision, aside from the high myopia eyes with the postoperative − 2.25 to -1.50 refraction. In high myopia cases with a target range of -1.50 to -0.75, individuals experienced a more favorable reduction in spectacle dependency across all distances compared to other postoperative SE groups. This suggests that other retained myopic corrections may be unsuitable for individuals with high myopia. With the target of -1.50 to -0.75 myopic diopters approach seems to improve near visual acuity to rectify the inadequacy near vision of EDOF IOLs.

Our study had certain limitations. Specifically, we did not obtain data on the defocus curve, mesopic contrast sensitivity, aberrometry results, and responses to subject questionnaires regarding visual symptoms. These aspects would be accurately assessed and addressed in future investigations. The retrospective study design was its innate shortcoming, as well as the comparatively smaller sample involved. Further study is needed to evaluate more axial myopia patients with EDOF IOL implantation prospectively.

## Conclusions

In summary, our current study demonstrates that the implantation of EDOF IOLs in eyes with axial lengths longer than 26 mm or shorter still resulted in satisfactory visual and refractive outcomes. Furthermore, retaining refractive diopters within the range of -1.50 to -0.75 in high myopia eyes may lead to better visual quality and increased spectacle independence.

## Data Availability

The datasets used and analyzed during the current study are available from the corresponding author on reasonable request.

## Abbreviations

EDOF IOL: extended depth of focus intraocular lens
AL: axial length
CDVA: corrected distance visual acuity
UDVA: uncorrected distance visual acuity
CS: contrast sensitivity
SE: spherical equivalent
HMC: high myopia combined with cataract patients
D: diopter
ACD: anterior chamber depth
CCC: continuous curvilinear capsulorhexis
PCO: posterior capsular opacification
UDVA: uncorrected distance visual acuity
UIVA: uncorrected intermediate visual acuity
UNVA: uncorrected near visual acuity
logMAR: logarithms of the minimal angle of resolution
OCCIs: opposite clear corneal incisions
